# Two Cases of Solitary Fibrous Tumor Involving Urinary Bladder and a Review of the Literature

**DOI:** 10.1155/2016/5145789

**Published:** 2016-10-04

**Authors:** Eduardo Yukio Tanaka, Vitor Bonadia Buonfiglio, Joao Padua Manzano, Renée Zon Filippi, Marcus Vinicius Sadi

**Affiliations:** ^1^Federal University of São Paulo, São Paulo, SP, Brazil; ^2^Hospital Israelita Albert Einstein, São Paulo, SP, Brazil

## Abstract

Solitary fibrous tumor (SFT) is a rare neoplasia of mesenchymal origin, initially described in visceral pleura and lately discovered to have ubiquitous distribution. SFT of the urogenital tract is uncommon and appears to have similar morphologic features and biologic behaviors as SFTs found elsewhere. We present two new cases of SFT of the bladder and review 22 similar cases published in the literature. Due to the general indolent behavior of these lesions, a complete but organ sparing surgical excision should be considered when technically feasible. Therefore, proper identification and characterization of SFT through morphological and immunohistochemical criteria on biopsy specimens are mandatory in the differential diagnosis from other more aggressive spindle-cell tumors, thus avoiding unnecessary radical surgery.

## 1. Introduction

Solitary fibrous tumor (SFT) is a rare neoplasia of mesenchymal origin, initially described in visceral pleura and lately found to have ubiquitous distribution [1]. Most tumors are benign and, contrary to previous beliefs, they do not derive from the mesothelium but rather from dendritic interstitial cells, which express CD34 and have generalized distribution in tissues, a feature that helps to recognize them in other organs [2, 3].

The urogenital tract involvement is very rare. We present two cases of SFT of the bladder and review 22 cases published in the literature to date.

## 2. Case Report 1

During a routine abdominal ultrasound examination, a bulging of bladder floor with discrete left ureteral dilatation was detected in an asymptomatic 60-year-old man. The patient's medical history was unremarkable. Clinical examination revealed an enlarged prostate of 40 cm^3^. A complete biochemical workup evaluation was normal and his PSA level was 2.1 ng/mL.

**Figure 1:**
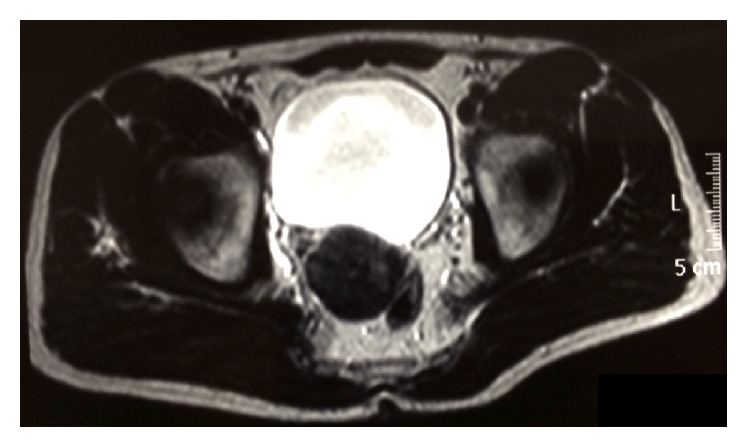
Magnetic resonance imaging T2-weighted imaging showed a mass of 5 cm in diameter on the posterior wall of the bladder.

Computed tomography (CT) revealed a well-delineated and homogeneous retrovesical solid mass that measured 5 × 3 cm in diameter (Figure 1).

Magnetic resonance imaging (MRI) confirmed the presence of this noninfiltrative solid tumor, between the rectum and the bladder with a thin capsule of low signal in T2.

A transrectal ultrasound needle guided biopsy was performed with a presumptive diagnosis of a pelvic sarcoma. The histology showed spindle-cell-shaped proliferation expressing CD34 antigen and vimentin. Immunochemistry was positive for Bcl-2 favoring the diagnosis of SFT.

Additional staging examinations, including chest X-ray and bone scan, did not reveal metastases. Colonoscopy shows no alteration.

Extirpative surgery through an infraumbilical midline incision was scheduled. The bladder and rectum could be well separated from the tumor and both organs were spared. The pathology report described an 8,0 × 6,0 tumor weighting 101 grams, with a regular surface and negative surgical margins. His postoperative recovery was uneventful.

**Figure 2:**
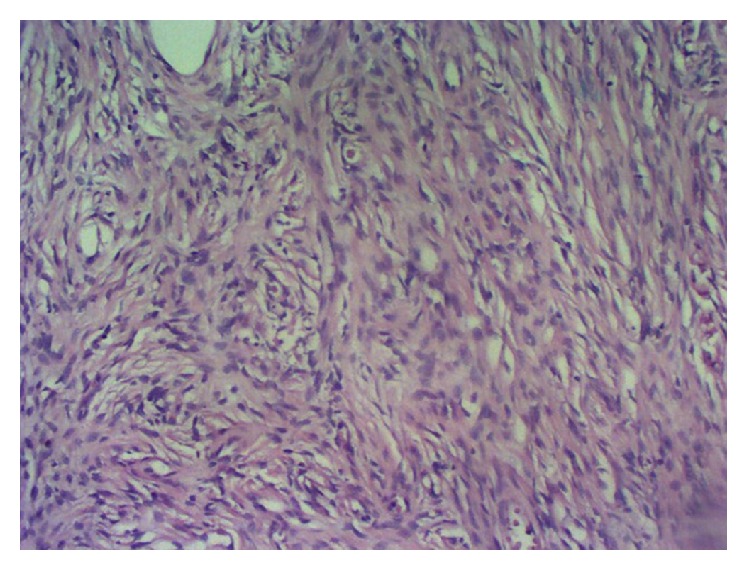
Hematoxylin and eosin stained section shows spindle-shaped cells.

**Figure 3:**
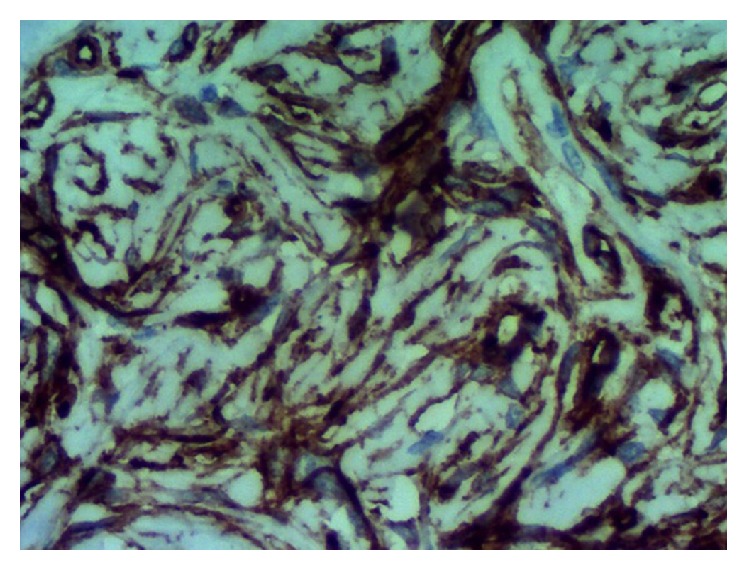
Immunohistochemistry of CD34.

**Figure 4:**
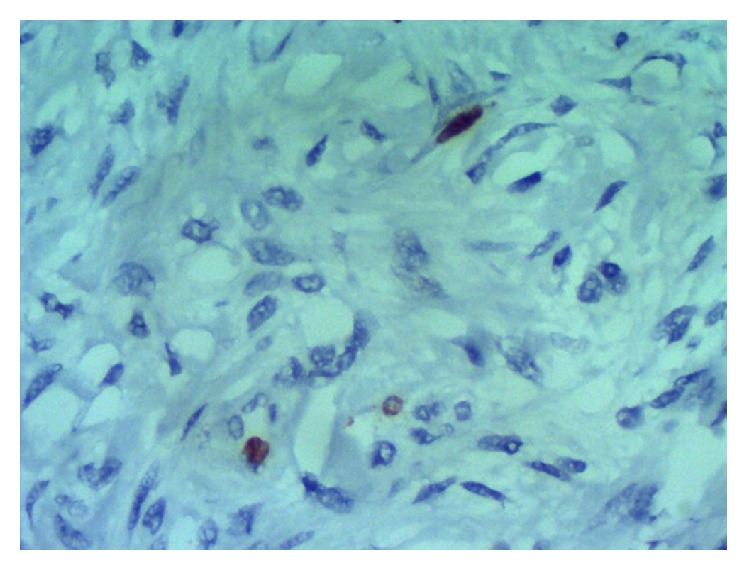
Immunohistochemistry of Ki-67.

**Figure 5:**
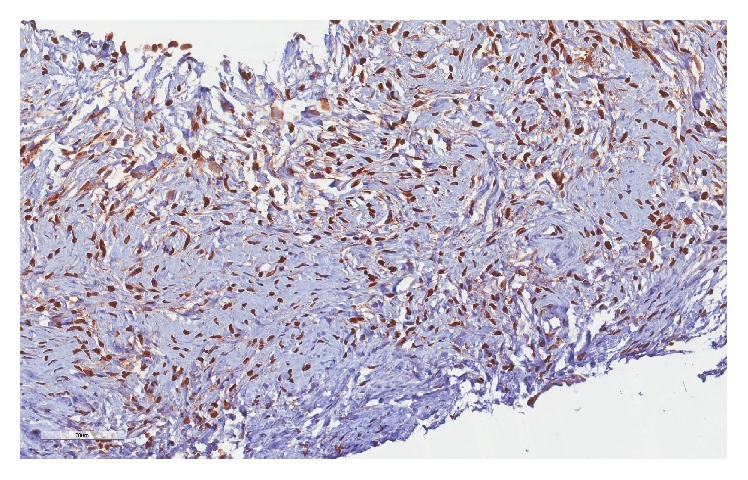
Diffuse nuclear expression of STAT6.

Immunohistochemistry demonstrated expression of CD34 (Figure 3), Ki-67 (Figure 4), and STAT6 (Figure 5) but no expression of ALK, EMA, S100, 1A4, HHF 35, CD117, and CD99. The tumor consisted of irregularly dispersed cells in a so-called patternless. The cells were rounded and spindle-shaped with little pleomorphism and few vesicular nuclei and were intermingled with thick collagen fiber bundles. In parts, cystic degenerative changes and vascular and pseudovascular formations with broad rims of hyalinized collagen were observed (Figure 2). No reactivity was detected using a CD117 (c-kit) antibody, consistent with the diagnosis of a SFT. The proliferation index (Ki-67) was less than 2% (Figure 4) suggesting a probable benign biologic behavior. Two years after surgery, the patient is well with no signs of tumor recurrence.

## 3. Case Report 2

A 60-year-old man with a rising PSA of 4,9 ng/mL was found to have a perivesical mass in the left side of bladder on transrectal ultrasound (US). An US guided biopsy of the prostate and bladder was performed simultaneously. The histopathology showed a prostate acinar adenocarcinoma Gleason Score 7(3 + 4) and a bladder hemangiopericytoma. The patient underwent a nerve-sparing retropubic radical and partial cystectomy to remove the perivesical bladder lesion during the same surgical procedure. Recovery was uneventful and the patient was discharged on the 3rd postoperative day.

The final pathology report showed prostate acinar adenocarcinoma with Gleason Score 7(3 + 4) of the right lobe (pT2a) and a well-circumscribed mass measuring 4,0 cm × 4,0 cm. The diagnosis of a bladder SFT was rendered based on the histopathologic and immunohistochemical findings consisting of short-spindled cells with meager amounts of eosinophilic cytoplasm without necrosis. The tumor cells were immunoreactive to CD34, CD99, c-kit, and Bcl-2 and they were negative for CD117 (c-kit), anaplastic lymphoma kinase (ALK-1), smooth muscle actin, desmin, cytokeratin, and S100 protein. Mitotic activity was <1 mitosis per 10 HPFs. After 10 years of follow-up, his PSA is undetectable and there are no signs of the SFT recurrence in the bladder.

## 4. Discussion

Solitary fibrous tumor (SFT) is a rare mesenchymal neoplasm that accounts for less than 2% of all soft-tissue tumors usually involving the pleura, pericardium, and peritoneum. Klemperer and Rabin reported the first five cases of primary SFT in 1931 [23]. For a long time, SFTs were described as “benign fibrous mesotheliomas” of the pleural cavity and these tumors were erroneously thought to be exclusively confined to the serosal surfaces, due to an assumed mesothelial origin [24]. It is now well established that SFTs are ubiquitous neoplasms with both pleural and extrapleural distribution [25–28]. These tumors have been reported to arise from many organs including orbit, meninges, paranasal sinuses and upper respiratory tract, thyroid, sublingual gland, lung, mediastinum, pericardium, gastrointestinal tract, liver, kidney, peritoneum, adrenal gland, spinal cord, ovary, uterine cervix/vagina, bladder, prostate, scrotum, testicular tunica vaginalis, skin, soft tissue, and periosteum. The first case involving the urinary tract was reported in 1997 [2].

SFT occurs equally in both sexes and the age of presentation varies from the second to sixth decade. Clinically it is a slow-growing, painless, well-delineated exophytic mass. Fewer than 5% of SFT presents with paraneoplastic syndromes such as hypoglycemia secondary to insulin-like growth factor [2]. In our review of 22 cases of SFT of the urinary bladder previously described in the English literature (Table 1), 36% of the patients had voiding difficulty, 32% had hematuria, 18% had incidental imaging finding, and 14% presented with lower abdominal discomfort.

Most tumors have a benign clinical course, although 10% to 20% may show aggressive behavior. The criteria for malignancy include increased cellularity, pleomorphism, increased mitotic activity (more than 4 mitoses on 10 high power fields), necrosis, and hemorrhage [2]. Factors associated with aggressive behavior include positive surgical margins, tumor size greater than 10 cm, and poor histology [1].

The diagnosis depends on histological and immunohistochemical examinations. The pathological analysis features proliferation of bland-looking spindle to oval epithelioid cells that form fascicles between collagen bundles with a prominent vasculature simulating hemangiopericytoma [29]. Hemangiopericytoma and SFT form a histologic spectrum of fibroblastic-type mesenchymal neoplasms with overlapping clinical, imaging, and cytopathologic features. Some SFTs are incorrectly characterized as hemangiopericytomas due to pericyte differentiation [30, 31]. The main differential diagnosis other than hemangiopericytoma includes sarcomas, leiomyomas, and inflammatory pseudotumors.

Immunohistochemistry shows positivity to Bcl-2, CD34 (90–95%), CD99 (70%), and vimentin. Cytokeratin AE1/AE3, CD31, and S100 protein are usually negative [2]. The nuclear expression of STAT6 protein was analyzed only in case 1, because during the data collection it was not possible to retrieve the sample of case 2. Recent studies have demonstrated that STAT6 immunohistochemistry is positive in up to 100% of SFTs [32]. Therefore, STAT6 has emerged as a highly sensitive marker for SFT that can reliably distinguish from its mimics.

A complete surgical excision with negative margins whenever feasible is the treatment of choice with a five-year overall survival approaching 100%. Due to its indolent behavior, a proper identification and characterization of SFT through morphological and immunohistochemical criteria could avoid misinterpreting these tumors as other more aggressive lesions, thus precluding an organ sparing approach.

According to our review, biopsy results were inconclusive in 20% (2/10) of the patients. Two cases reported increased mitotic activity and two others expressed IGF-II [16, 33]. The only radical treatment was reported by Lam et al. who performed a radical cystectomy, left nephrectomy, and an ileal conduit due to a 7 cm bladder mass extending into the left lower ureter causing hydronephrosis but this patient did not have a previous biopsy of the bladder lesion [7].

## 5. Conclusion

Most SFTs of the bladder have an indolent course and a favorable prognosis. The treatment of choice is complete resection with negative margins. Factors associated with aggressive behavior include positive surgical margins, tumor size greater than 10 cm, and poor histology. We advise urologists to consider the diagnosis of SFT when biopsy specimens have spindle-cell neoplasia present thus promoting the possibility of a more conservative surgical approach.

## Figures and Tables

**Table 1:** Reported 24 cases of SFT originating in urinary bladder.

	Authors	Age (years)	Sex	Symptoms	Tumor size (cm)	Treatment	Follow-up (months)
1	Bainbridge et al. [4]	50	F	Increase in urinary frequency	5.2	Transurethral resection of the bladder	18
2	Bainbridge et al. [4]	42	M	Pelvic pressure	20	Wide excision	6
3	Westra et al. [5]	67	M	Voiding difficulty: incidental cystoscopy finding during TURP	4	Cystoprostatectomy (completely excised)	9
4	Westra et al. [5]	67	M	Incidental MRI finding (prostate cancer surveillance)	NA	Transurethral resection of the bladder	1
5	Corti et al. [6]	50	M	Pelvic pain, dysuria, and urinary bleeding	6.5	Cystoprostatectomy (completely excised)	18
6	Lam et al. [7]	44	M	Painless gross hematuria	7	Excision (radical cystectomy, left total nephrectomy)	NA
7	Huang et al. [8]	70	M	Urinary frequency, urgency, tenesmus	19	Excision	12
8	Kim et al. [9]	56	M	Voiding difficulty	12	Wide excision	12
9	Leite et al. [10]	60	M	Incidental MRI finding (prostate cancer screening)	3.2	Complete tumor excision and radical prostatectomy	11
10	Ishikawa et al. [11]	64	M	Voiding difficulty	12.5	Excision	3
11	Tsurukawa et al. [12]	61	M	Recurrent hypoglycemic attack and lower abdominal discomfort	15	Excision	NA
12	Tzelepi et al. [13]	59	F	Hematuria	8.5	Radical cystectomy with an orthotopic ileal bladder substitution	77
13	Heinzelbecker et al. [14]	24	F	Hematuria	8.5	TURBT and partial cystectomy	24
14	Wang et al. [15]	50	M	Hematuria	8	Wide tumor excision	9
15	Martín and Fernández [2]	59	M	Voiding difficulty	3.5	Excision	24
16	Bruzzone et al. [16]	74	M	Chills, diaphoresis, and acute abdominal pain with hematuria	10	Excision	NA
17	Pata et al. [17]	76	M	Low abdominal pain, acute urine retention	17	Wide excision, bladder preservation	60
18	Wang et al. [18]	72	M	Incidental MRI finding	0.85	Transurethral resection	16
19	Cheng et al. [19]	67	M	Low abdominal pain	16	Partial cystectomy and segmental resection of the intestine	18
20	Seike et al. [20]	41	F	Incidental TC finding	5.2	Transurethral resection	3
21	Otta et al. [21]	78	M	Hematuria	NA	Transurethral resection	36
22	Spairani et al. [22]	60	M	Voiding difficulty	9	Partial cystectomy	11
23	Our case	60	M	Incidental US finding	8	Wide excision, bladder preservation	24
24	Our case	60	M	Incidental US finding	4	Partial cystectomy and radical prostatectomy	120
